# The Recently Identified Isoleucine Conjugate of *cis*-12-Oxo-Phytodienoic Acid Is Partially Active in *cis*-12-Oxo-Phytodienoic Acid-Specific Gene Expression of *Arabidopsis thaliana*

**DOI:** 10.1371/journal.pone.0162829

**Published:** 2016-09-09

**Authors:** Monika D. Arnold, Cornelia Gruber, Kristýna Floková, Otto Miersch, Miroslav Strnad, Ondřej Novák, Claus Wasternack, Bettina Hause

**Affiliations:** 1 Department of Cell and Metabolic Biology, Leibniz Institute of Plant Biochemistry, Halle (Saale), Germany; 2 Laboratory of Growth Regulators, Centre of the Region Haná for Biotechnological and Agricultural Research, Institute of Experimental Botany AS CR & Palacký University, Olomouc, Czech Republic; 3 Department of Molecular Signal Processing, Leibniz Institute of Plant Biochemistry, Halle (Saale), Germany; Estacion Experimental del Zaidin, SPAIN

## Abstract

Oxylipins of the jasmonate family are active as signals in plant responses to biotic and abiotic stresses as well as in development. Jasmonic acid (JA), its precursor *cis*-12-oxo-phytodienoic acid (OPDA) and the isoleucine conjugate of JA (JA-Ile) are the most prominent members. OPDA and JA-Ile have individual signalling properties in several processes and differ in their pattern of gene expression. JA-Ile, but not OPDA, is perceived by the SCF^COI1^-JAZ co-receptor complex. There are, however, numerous processes and genes specifically induced by OPDA. The recently identified OPDA-Ile suggests that OPDA specific responses might be mediated upon formation of OPDA-Ile. Here, we tested OPDA-Ile-induced gene expression in wild type and JA-deficient, JA-insensitive and JA-Ile-deficient mutant background. Tests on putative conversion of OPDA-Ile during treatments revealed only negligible conversion. Expression of two OPDA-inducible genes, *GRX480* and *ZAT10*, by OPDA-Ile could be detected in a JA-independent manner in Arabidopsis seedlings but less in flowering plants. The data suggest a bioactivity *in planta* of OPDA-Ile.

## Introduction

Oxylipins of the jasmonate family are lipid derived signals with functions in numerous plant responses to biotic and abiotic stress as well as in development. The most bioactive compound is a specific enantiomer of the isoleucine conjugate of JA, the (+)-*7-iso*-JA-Ile [1]. This compound is formed by the jasmonoyl-isoleucine conjugate synthase (JAR1) [2] and acts as the ligand of the SCF^COI1^ JAZ co-receptor complex [3–5] which has been analysed also upon crystallization [6]. The co-receptor complex is consisting of an F-box protein called CORONATINE INSENSITIVE 1 (COI1), a member of an Skp1/Cullin/F-box complex with E3 ubiquitin ligase activity, and its targets, the JASMONATE ZIM DOMAIN (JAZ) proteins. The ligand (+)-*7-iso*-JA-Ile acts as molecular glue that enables the interaction between COI1 and JAZ proteins leading to proteasomal degradation of JAZ (for review see [7]). In pull-down experiments and yeast-two-hybrid assays it was shown that the receptor complex binds exclusively and specifically JA-Ile, but not JA or its methyl ester JAMe [3]. The bioactivity of JA and JAMe could be experimentally proven and explained by conjugation of JA to JA-Ile by JAR1 [2] and in case of JAMe by transgenic approaches showing requirement for cleavage to JA and subsequent conjugation to JA-Ile [8]. In contrast, there was no binding of OPDA to the co-receptor complex in pull-down experiments [1, 3] suggesting JA-Ile-specific signalling mediated by COI1. There are, however, numerous processes and genes which are specifically activated by OPDA in a JA-Ile- and COI1-independent manner (cf. reviews of [7, 9–11]. Among them are seed germination [12], seed dormancy [13], embryo development in tomato [14], drought stress responses in *Arabidopsis thaliana* and tomato [15], local defence responses of tomato [16] or tendril coiling in *Bryonia dioica* and *Phaseolus vulgaris* [17]. Expression analyses showed distinct sets of genes specifically expressed by OPDA [18, 19]. Furthermore, OPDA but not JA occurs in *Marchantia polymorpha* [20] and *Physcomitrella patens* [21, 22] exhibiting specific functions in defence and development. Due to these OPDA-specific processes as well as the above mentioned lack of binding of OPDA to the co-receptor complex active in JA-Ile perception, there is the open question on OPDA perception. So far, there is no hint on an OPDA-receptor. One possibility for OPDA perception is its binding by cyclophilin 20–3 which leads to enhanced redox capacity followed by expression of OPDA-induced genes [23]. Another possibility is that OPDA is not active per se but might be active upon conjugation with isoleucine to OPDA-Ile. This compound, however, could not be identified in *P*. *patens* even there is no formation of JA but OPDA accumulation including OPDA-specific responses [21]. Only recently, OPDA-Ile could be identified in flowering *A*. *thaliana* plants [24] rising the question on bioactivity of OPDA-Ile in this plant.

Here, we addressed the question on OPDA-Ile bioactivity by recording expression of two genes known to be induced by OPDA. Among many genes, *ZAT10* and *GRX480* were shown to be specifically induced by OPDA [18, 23]. *ZAT10* encodes a salt-tolerance zinc-finger protein and acts as transcription factor in the plant’s response to various abiotic stresses[18], whereas *GRX480* encodes a GLUTAREDOXIN and is considered as candidate for an OPDA-associated transcription regulator [23]. Therefore, we have been chosen these two genes to prove putative transcript accumulation by OPDA-Ile treatment compared to that of OPDA in different genetic background. The well-known Arabidopsis mutants *opr3* and *jar1*, affected in the OPDA reductase3 of JA biosynthesis and in the conjugation of JA to JA-Ile, respectively, were used to record OPDA-specific responses without interference with JA/JA-Ile responses. Analytical proof on stability and conversion, respectively, of OPDA-Ile during treatment in wild type and mutant background showed its stability. The genes *GRX480* and *ZAT10* were significantly expressed by treatment of seedlings but less of flowering plants with OPDA-Ile suggesting a role of this compound as a signal *in planta*.

## Materials and Methods

### Plant material and cultivation

*A*. *thaliana* ecotypes Col-0, Col-5 and Wassilewskija (WS) were used as control wild-type species. The JA-deficient mutant *OPDA reductase3* (*opr3*) (in the WS background, [25]) was kindly provided by J. Browse, the JA-Ile-deficient mutant *JA-resistant1* (*jar1*) (in the Col-0 background, [2]) was a gift from P.E. Staswick, and the JA-insensitive mutant *coronatine-insensitive1-16* (*coi1-16*) (in the Col-5 background, [26]) was obtained from Nottingham Arabidopsis Stock Centre [27]. All seeds were surface-sterilized in 70% ethanol and 3% H_2_O_2_ for 6 min, washed extensively in sterile distilled water and cold treated at 4°C for 3 d. The surface-sterilized seeds were germinated for 9 or 10 days at 21°C in a growth chamber (16 h light/8 h dark) in wells of a 12-well suspension culture plate containing 2 ml Murashige and Skoog medium and 0.2% (w/v) sucrose per well. To obtain flowering plants, seedlings were transferred to soil and grown in a growth chamber under long day conditions as described [24].

### Treatments

OPDA and OPDA-Ile were synthesized enzymatically according to a modification of the procedure reported by [28] and as described in [24], respectively. [^2^H_5_]OPDA-Ile was prepared by the method of [29] using [^2^H_5_]OPDA. Seedlings were treated with OPDA or OPDA-Ile dissolved in acetonitrile (0.87% [v/v] and 0.56 [v/v] final concentration, respectively) and supplied with 0.025%(v/v) Silvet L-77 by exchanging the liquid growth medium. As control, seedlings were treated with 0.87% or 0.56% acetonitrile (+ 0.025%(v/v) Silvet L-77). Rosette leaves of flowering plants were carefully dissected from the plants and floated on the respective solutions. At the indicated time points, plant material (25 mg of fresh weight) was flash-frozen in liquid nitrogen and stored at -80°C until further use.

### Feeding experiments with labelled OPDA-Ile

Ten-day-old Arabidopsis seedlings (Col-0) were incubated with liquid medium containing 30 μM [^2^H_5_]OPDA-Ile for 30 min. The samples (25 mg of whole seedlings) were extracted and purified by solid phase extraction (SPE) as described in [24]. Metabolization of OPDA-Ile shown as concentration of labelled metabolites was measured using LC-MS/MS, with the MRM transitions corresponding to labelled compounds after correction for natural isotope abundances.

### Quantification of OPDA, OPDA-Ile, JA and JA-Ile

OPDA, JA, and JA-Ile, were quantified simultaneously using a standardized ultraperformance liquid chromatography–tandem mass spectrometry (UPLC-MS/MS)-based method according to [30] using [^2^H_5_]OPDA, [^2^H_6_]JA, and [^2^H_2_]JA-Ile as internal standards. Quantification of OPDA-Ile simultaneously to OPDA, JA and JA-Ile was done according to [24] using [^2^H_5_]OPDA-Ile as additional internal standard. Synthesis of [^2^H_5_]OPDA, [^2^H_6_]JA, and [^2^H_2_]JA-Ile was performed as described [28, 29].

### Quantitative RT-PCR analysis

RNA isolation and determination of transcript accumulations of *AtGRX480* (At1g28480) and *AtZAT10* (At1g27730) were done as described by [31] using *AtPP2A* (At1g10430) as a constitutively expressed control [32]. The following primers were used: *AtGRX480*: for 5’-TGATTGTGATTGGACGGAGA-3’, rev 5’-TAAACCGCCGGTAACTTCAC-3’, *AtZAT10*: for 5’-AGGCTCTTACATCACCAAGATTAG-3‘, rev 5‘-TACACTTGTAGCTCAACTTCTCCA-3’, *AtPP2A*: for 5’-AGACAAGGTTCACTCAATCCGTG-3’, rev 5’-CATTCAGGACCAAACTCTTCAGC-3’. All assays were performed with three technical replicates, and three biologically independent samples were used. ΔCt-values were calculated by subtracting Ct-values of the target gene from the Ct-value of the constitutively expressed *AtPP2A* gene. Comparative expression levels were calculated as 2^ΔCt^.

## Results

**Fig 1:**
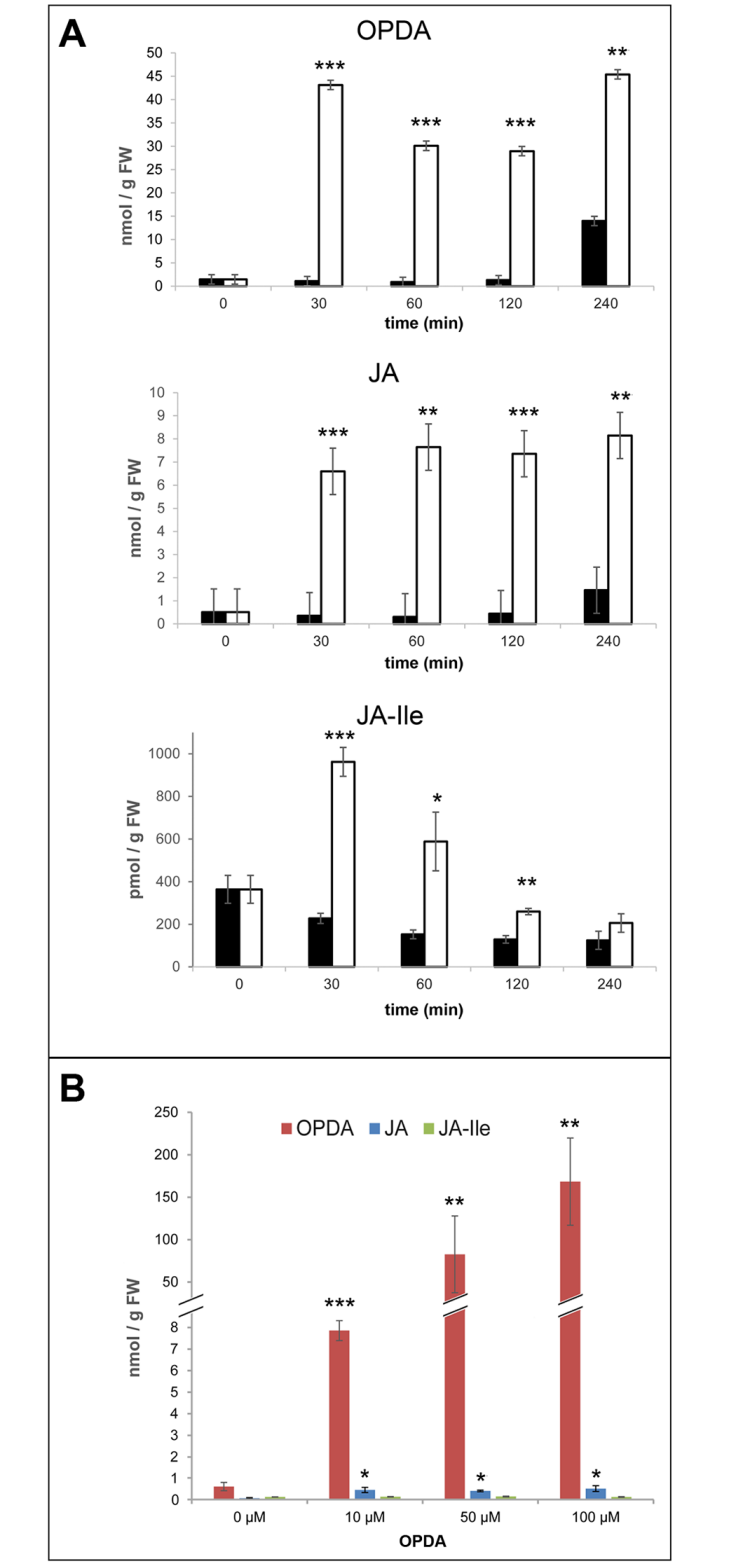
Conversion of 12-oxophytodienoic acid (OPDA) in leaves of *Arabidopsis thaliana* ecotype WS (wild type, A) and *opr3* (B). A, Leaves of 5-week-old WS plants grown under short-day conditions were floated on water (control, black bars) or on 50 μM OPDA for the time periods indicated. Levels of OPDA, jasmonic acid (JA) and JA-Ile are presented. B, Leaves of 5-week-old *opr3* plants grown under short-day conditions were floated on solutions containing different concentrations of OPDA for 30 min. Levels of OPDA (red bars), JA (blue bars) and JA-Ile (green bars) are presented. Contents of OPDA, JA and JA-Ile were determined by LC-MS according to [30]. Each value is represented by the mean of three independent biological replicates ± SD. Treatments and controls were pairwise compared by the Student’s t-test, *p≤0.05, **p≤0.01, ***p≤0.001.

As prerequisites for testing OPDA- and OPDA-Ile-induced gene expression, incubation time, concentrations, and stability of applied compounds were determined. Initial kinetics on levels of JA, JA-Ile and OPDA upon application of 50 μM OPDA to leaves of WS plants revealed for all compounds high levels already after 30 min of incubation (Fig 1A). Levels of OPDA and JA remained high in the following incubation period, but levels of JA-Ile declined already after 30 min of treatment. Control treatments done by application of solvent did not cause alterations in endogenous hormone contents within two hours of treatment (Fig 1A, black bars). Therefore, treatment of plant materials with OPDA for 30 min was selected for the following experiments. Additionally, it was checked whether *opr3* is impaired in conversion of OPDA to JA and JA-Ile, since it was reported recently that *opr3* is under some conditions not a null mutant [33]. Upon application of 10 μM, 50 μM and 100 μM OPDA for 30 min, OPDA levels in *opr3* leaves increased up to 150 nmol/g FW, whereas JA and JA-Ile levels remained constantly low (Fig 1B). These results revealed that the *opr3* mutant represents a reliable control for OPDA-specific processes, since conversion of OPDA to JA and JA-Ile was not detectable as described formerly by [25]. Moreover, high levels of OPDA revealed sufficient uptake of OPDA by the plant material. Therefore, 30 μM OPDA or OPDA-Ile were used in the following experiments.

**Fig 2:**
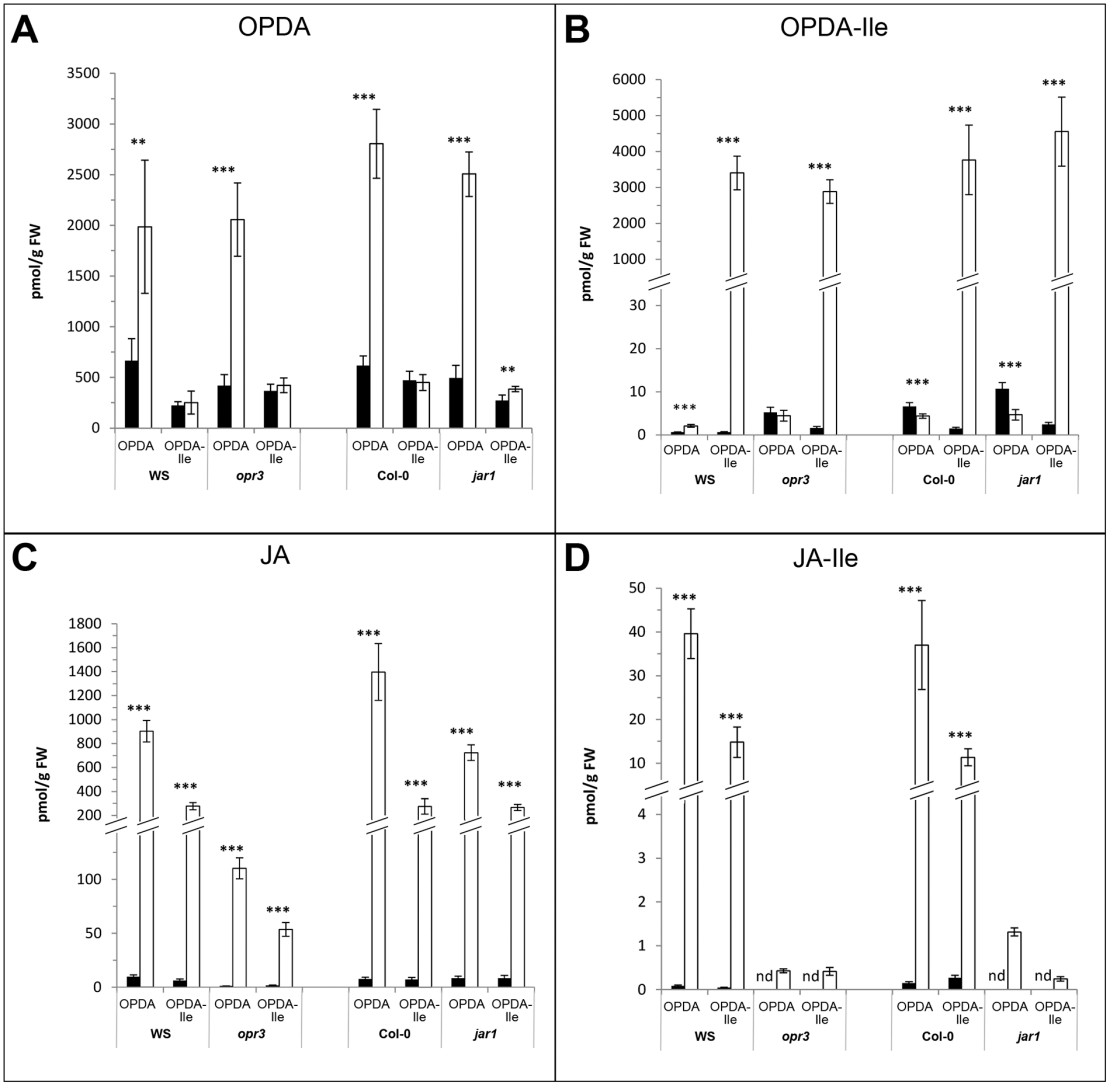
Levels of OPDA (A), OPDA-Ile (B), JA (C) and JA-Ile (D) in seedlings of *Arabidopsis thaliana* WS, *opr3*, Col-0 and *jar1* after treatment with OPDA or OPDA-Ile. 10-days-old seedlings grown in liquid culture were treated with 30 μM OPDA or 30 μM OPDA-Ile for 30 min (white bars). Two independent controls (black bars) were performed by treatment with bi-distilled H_2_O containing 0.87% [v/v] and 0.56% [v/v] acetonitrile, respectively. Compounds were quantified according to [24]. Each value is represented by the mean of five independent biological replicates ± SD. Treatments and controls were pairwise compared by the Student’s t-test, *p≤0.05, **p≤0.01, ***p≤0.001.

Additionally, stability of applied OPDA-Ile during treatment was checked by treatment of 10-days-old Col-0 seedlings with 30 μM ^2^H_5_-OPDA-Ile for 30 min. 2852.9 +/- 333.8 pmol per g FW were detected within the plant material indicating sufficient uptake. Only 436.8 +/- 75.8 pmol per g FW were found as ^2^H_5_-OPDA which suggests a marginal cleavage of the applied compound. This value corresponds to the amount of OPDA detected upon treatment of 10-days-old Col-0 seedlings with 30 μM unlabelled OPDA-Ile for 30 min (cf. below Fig 2A).

To check in more detail putative conversion of OPDA and OPDA-Ile in later gene expression analysis, the levels of OPDA, OPDA-Ile, JA and JA-Ile were recorded in untreated and treated 10-days-old seedlings of both WTs (Col-0 and WS) and both mutants (*opr3* and *jar1*) (Fig 2). As expected, the applied OPDA and OPDA-Ile, respectively, were detected at high levels in WTs and mutants at the chosen time of treatment (30 min, Fig 2A and 2B). A minor conversion of OPDA to OPDA-Ile was detected in WS (Fig 2B), whereas lower levels of OPDA-Ile were found upon OPDA treatment in all other genotypes. Also a cleavage of OPDA-Ile is highly improbable, since only a minor amount of OPDA and only in the *jar1* mutant background was found upon treatment with OPDA-Ile (Fig 2A). As expected, JA was formed upon application of OPDA in both WTs, but much less (4-times) after application of OPDA-Ile (Fig 2C). The mutant *opr3* showed significant increase in JA levels upon treatment with OPDA or OPDA-Ile. These levels were, however, 10-fold less than in other genotypes. Most importantly, there was no detectable level of JA-Ile in the *opr3* and *jar1* mutant upon treatment with both compounds. These data on putative conversion of OPDA and OPDA-Ile suggest that any response to each compound in terms of gene expression in *opr3* or *jar1* mutant background can be referred to OPDA and OPDA-Ile, respectively, but not to JA-Ile. Similar analyses were performed for leaves of six-week-old flowering plants (S1 Fig). Most of the above mentioned alterations in levels upon treatment with OPDA and OPDA-Ile in the different genetic background reflect the pattern found for seedlings, but are less clear due to a low uptake of OPDA and OPDA-Ile and the high basal levels of OPDA in adult leaves. Moreover, leaves of flowering plants of all genotypes contained much higher basal levels of OPDA which appeared to be 40-fold higher than in seedlings. Furthermore, basal JA levels in all genotypes except *opr3* are greatly enhanced in comparison to seedlings (Fig 2). Similarly to seedlings, however, JA-Ile could not be detected in *opr3* and *jar1* mutant leaves upon treatment with OPDA or OPDA-Ile.

Based on these prerequisites, gene expression analyses were performed for *GRX480* and *ZAT10* upon treatment with OPDA and OPDA-Ile, respectively, in each genetic background described above. To confirm initially that the selected OPDA-induced genes are expressed COI1-independently, expression analysis of *GRX480* in seedlings of the *opr3* mutant as well as the JA-insensitive mutant *coi1-16* and the corresponding WTs WS and Col-5 was performed (S2 Fig). The data in terms of fold-change of expression indicated a response already after 30 min of treatment in all genotypes, which did not change tremendously up to four hours. Most importantly, however, there was no significant difference in induction between the mutants and their respective wild types. The data clearly show that (i) OPDA itself is inducing *GRX480* gene expression, and (ii) this induction does not depend on COI1.

**Fig 3:**
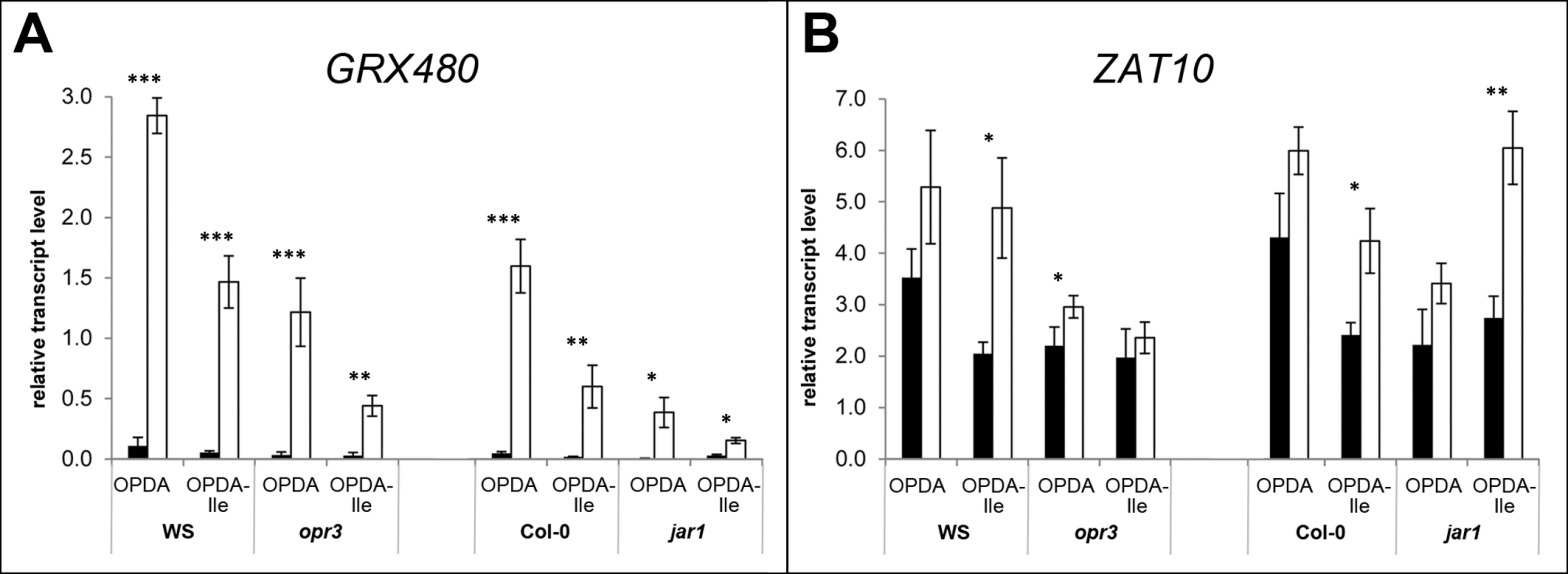
Relative transcript levels of *GRX480* (A) and *ZAT10* (B) in seedlings of *Arabidopsis thaliana* WS, *opr3*, Col-0 and *jar1* after treatment with OPDA or OPDA-Ile. 10-days-old seedlings grown in liquid culture were treated with 30 μM OPDA or 30 μM OPDA-Ile for 30 min (white bars). Two independent controls (black bars) were performed by treatment with bi-distilled H_2_O containing 0.87% [v/v] and 0.56% [v/v] acetonitrile, respectively. Relative transcript levels were quantified by qRT-PCR using *AtPP2A* as reference. Each value is represented by the mean of three independent biological replicates ± SD. Treatments and controls were pairwise compared by the Student’s t-test, *p≤0.05, **p≤0.01, ***p≤0.001.

To analyse the induction of *GRX480* expression by OPDA-Ile, 10-days-old seedlings of WS, *opr3*, Col-0 and *jar1* were treated with 30 μM OPDA or OPDA-Ile for 30 min (Fig 3A). Here, significantly elevated transcript levels were found in seedlings of all genotypes upon OPDA treatment and upon treatment with OPDA-Ile. This increase, however, was higher by OPDA treatment than by OPDA-Ile treatment. These data suggest that both compounds can induce *GRX480* expression in a JA-independent manner, although treatment with OPDA is more effective than that with OPDA-Ile. This was visible also in leaves of flowering plants, in which *GRX480* transcript levels were increased upon OPDA treatment, but not upon treatment with OPDA-Ile in (S3A Fig).

To substantiate this observation, identical experiments were performed for another OPDA-responsive gene, *ZAT10* (Figs 3B and S3B). In 10-days-old seedlings, a significantly elevated amount of *ZAT10* transcript was detected in the WS and Col-0 upon treatment with OPDA-Ile indicating response of seedlings to OPDA-Ile in terms of *ZAT10* transcripts (Fig 3B). Most importantly, seedlings of the *jar1* mutant, which form only residual amount of OPDA-Ile upon treatment with OPDA (Fig 1B) showed the highest *ZAT10* transcript accumulation upon treatment with OPDA-Ile, but not significantly enhanced levels upon treatment with OPDA. In the *opr3* mutant, no significantly altered expression of *ZAT10* could be detected. Moreover, there was no significantly altered expression of *ZAT10* in leaves of six-week-old flowering plants of all genotypes upon treatment with OPDA or OPDA-Ile (S2A Fig). Although the induction of *ZAT10* expression by OPDA and OPDA-Ile was relatively low in comparison to *GRX480*, all these data suggest that in the absence of active JA-Ile (Fig 2D), exogenous OPDA-Ile can alter expression of genes known to be induced by OPDA.

## Discussion

A new concept of JA-Ile perception was established in 2007. This concept includes the SCF^COI1^ complex with COI1 as the JA-Ile-specific F-box protein and JAZ proteins as repressors. Pull-down experiments revealed that the SCF^COI1^-JAZ co-receptor complex binds specifically JA-Ile via the interacting JAZ and COI1 proteins, but not JA, JA-Me or OPDA [3, 34]. Inspection of more than 40 different JA and OPDA derivatives on activity in COI1–JAZ interaction analyses revealed no activity for OPDA and OPDA-Ile [1]. The specific binding of JA-Ile to the receptor complex was confirmed by crystallization [6]. As introduced, however, there are several OPDA-specific responses in defence and development raising the question on OPDA perception. Another COI1-independent SCF complex may occur [35]. To the best of our knowledge, however, genetic screens to pick up a gene involved in OPDA perception were unsuccessful so far.

The absolute requirement of conjugation of JA with isoleucine for its bioactivity [1–3, 34] suggests the possibility that a similar scenario may occur in case of OPDA-specific responses. Over the last two decades simultaneous accumulation of OPDA and JA/JA-Ile was described for numerous stress responses and developmental processes. OPDA-Ile was, however, not detected even in plants with JA-deficiency and exclusive accumulation of OPDA as reported for *P*. *patens* and *M*. *polymorpha* [20, 21]. Recently, OPDA-Ile was identified as a minor constituent of flowering Arabidopsis plants [24] rising the question on its bioactivity. Upon wounding, there was a significant increase in OPDA-Ile levels in leaves of flowering plants of the wild types WS and Col-0 and the *opr3* mutant, but not in the *jar1* mutant [24]. Therefore, we analysed putative bioactivity of OPDA-Ile in these mutants and their corresponding wild types. The analysis was performed in terms of expression of genes such as *GRX480* and *ZAT10* known to be expressed in response to OPDA [18, 23]. Expression analysis was done upon application with the selected concentration of 30 μM and time of 30 min (Fig 1). Additionally, the transcript levels of *GRX480* were checked upon OPDA application in the *coi1-16* mutant background and showed unequivocally the COI1-independend expression of *GRX480* (S2 Fig).

Based on these prerequisites, putative conversion of the applied OPDA and OPDA-Ile, respectively, was checked in seedlings of the WT WS and Col-0 as well as the JA-deficient mutant *opr3* and the JA-Ile-deficient mutant *jar1* (Fig 2). Both applied compounds were detected at high levels in both WTs and both mutants indicating a sufficient uptake within the 30 min of treatment. A cleavage of OPDA-Ile seems to be negligible, since only a minor increase in OPDA content was detected upon OPDA-Ile application (Fig 2A), which corresponds well to the amount ^2^H_5_-OPDA formation upon application of ^2^H_5_-OPDA-Ile under identical conditions. Furthermore, there was only minor conversion of OPDA to OPDA-Ile detected only in WS, but not in all other genotypes (Fig 2B). Whether this conversion takes place via JAR1 enzyme activity has to be proven. So far substrate specificity tests for JAR1 did not included OPDA [36]. The JA-Ile deficiency, however, recorded after OPDA application in the *jar1* mutant support the role of JAR1 in formation of JA-Ile upon treatment with OPDA. Since OPDA-Ile could not be detected in wounded leaves of *jar1* [24], it is tempting to speculate that JAR1 might be also involved in conjugation of Ile with OPDA.

Assuming that OPDA released from OPDA-Ile is metabolized rapidly to JA, cleavage of OPDA-Ile could nevertheless be possible. As expected, JA was formed in both WTs and *jar1* upon application of OPDA, but much less after application of OPDA-Ile (Fig 2C). Since for JA generation cleavage of the applied OPDA-Ile to OPDA has to be assumed, this low amount of JA upon treatment with OPDA-Ile may reflect such a cleavage followed by conversion to JA (Fig 2C and 2D). In the *opr3* mutant, only a minor amount of JA compared to the wildtype was detected upon application of OPDA and OPDA-Ile (Fig 2C). Such minor levels of JA even in the mutant where the OPR3 is affected has been repeatedly detected [37]. This compound, however, might represent the biological inactive stereoisomer *cis-*JA produced by the action of OPR1 and OPR2 enzymes present in the *opr3* mutant [25, 38, 39]. Most importantly, however, there was no detectable level of JA-Ile in the *opr3* and *jar1* mutant upon treatment with OPDA and OPDA-Ile indicating JA-Ile deficiency in both mutants (Fig 2D). These data strongly suggest that gene expression analysed upon application of OPDA or OPDA-Ile reflects responses to these compounds without interference by JA or JA-Ile. A similar pattern of levels was recorded for leaves of flowering plants (S1 Fig), but the higher basal levels of OPDA and JA in untreated tissues as well as the insufficient uptake of OPDA and OPDA-Ile (S1 Fig) did not allow a detailed evaluation of data. Altogether, the negligible cleavage of OPDA-Ile and minor conversion of OPDA to OPDA-Ile during treatment indicate that any putative bioactivity of applied OPDA-Ile in terms of gene expression can be regarded as activity of OPDA-Ile *per se*.

Expression analyses with seedlings showed for *GRX480* a significant induction by both compounds, OPDA and OPDA-Ile in each genetic background (Fig 3A).The induction by OPDA-Ile was, however, less than by OPDA. This contrasts to the results obtained for expression analysis done for *ZAT10*, which was induced clearly upon OPDA-Ile treatment but not upon OPDA treatment in all genotypes except *opr3* (Fig 3B). Expression of *GRX480* and *ZAT10* upon OPDA treatment was detected previously [18, 23]. Moreover, it was shown that OPDA application not only alters gene expression pattern [19] but also causes a dramatic alteration of the protein pattern in Arabidopsis, preferentially enzymes such as glutathione-S-transferases and other enzymes involved in redox homeostasis including conjugation of OPDA to these enzymes [40]. In all these data, however, putative conversion of the applied OPDA during treatment into JA and JA-Ile was not checked, although the use of *opr3* for gene expression studies pointed to the OPDA specificity. Here, we showed for the first time that OPDA-Ile is inducing OPDA-specific responses not only in wild type plants, but also in JA-deficient and JA-Ile-deficient mutant background indicating that OPDA-Ile is biologically active. This holds true for plants of different developmental stages, such as seedlings and leaves of flowering plants. Biological activity of OPDA-Ile may occur in nature, however, only under specific conditions, since OPDA-Ile was found exclusively in wounded leaves of flowering plants [24].

## Supporting Information

S1 Fig Levels of OPDA (A), OPDA-Ile (B), JA (C) and JA-Ile (D) in leaves of flowering *Arabidopsis thaliana* WS, *opr3*, Col-0 and *jar1* after treatment with OPDA or OPDA-Ile. Leaves of plants grown for 6 weeks under long-day conditions were floated on 30 μM OPDA or 30 μM OPDA-Ile for 30 min (white bars). Two independent controls (black bars) were performed by treatment with bi-distilled H_2_O containing 0.87% [v/v] and 0.56% [v/v] acetonitrile, respectively. Compounds were quantified according to Floková et al. (2016). Each value is represented by the mean of five independent biological replicates ± SD. Treatments and controls were pairwise compared by the Student’s t-test, *p≤0.05, **p≤0.01, ***p≤0.001.
(TIF)

S2 Fig Transcript accumulation pattern of *GRX480* in seedlings of WS and *opr3* (A) as well as Col-5 and *coi1-16* (B) after treatment with OPDA. 9-days-old seedlings grown in liquid culture were treated with 50 μM OPDA for the time periods indicated. Controls treated with the solvent acetonitrile (final concentration 0.87% [v/v]) for the same time periods showed constant transcript levels. Fold-change of relative transcript accumulation (ΔΔCt) was determined by qRT-PCR using *AtPPA2* as reference and setting t = 0 to 1. Each value is represented by the mean of three independent biological replicates ± SD. Mutants and their respective wild type were pairwise compared by the Student’s t-test and revealed no significant differences.
(TIF)

S3 Fig Relative transcript levels of *GRX480* (A) and *ZAT10* (B) in leaves of *Arabidopsis thaliana* WS, *opr3*, Col-0 and *jar1* after treatment with OPDA or OPDA-Ile. Leaves of plants grown for 6 weeks under long-day conditions were floated on 30 μM OPDA or 30 μM OPDA-Ile for 30 min (white bars). Two independent controls (black bars) were performed by treatment with bi-distilled H_2_O containing 0.87% [v/v] and 0.56% [v/v] acetonitrile, respectively. Relative transcript levels were quantified by qRT-PCR using *AtPP2A* as reference. Each value is represented by the mean of three independent biological replicates ± SD. Treatments and controls were pairwise compared by the Student’s t-test, *p≤0.05, **p≤0.01, ***p≤0.001.
(TIF)
