# Supplementary figures and images for: The Recently Identified Isoleucine Conjugate of cis-12-Oxo-Phytodienoic Acid Is Partially Active in cis-12-Oxo-Phytodienoic Acid-Specific Gene Expression of Arabidopsis thaliana
Source: PLoS One. 2016 Sep 9;11(9):e0162829. doi: 10.1371/journal.pone.0162829 (PMC5017875; doi:10.1371/journal.pone.0162829)

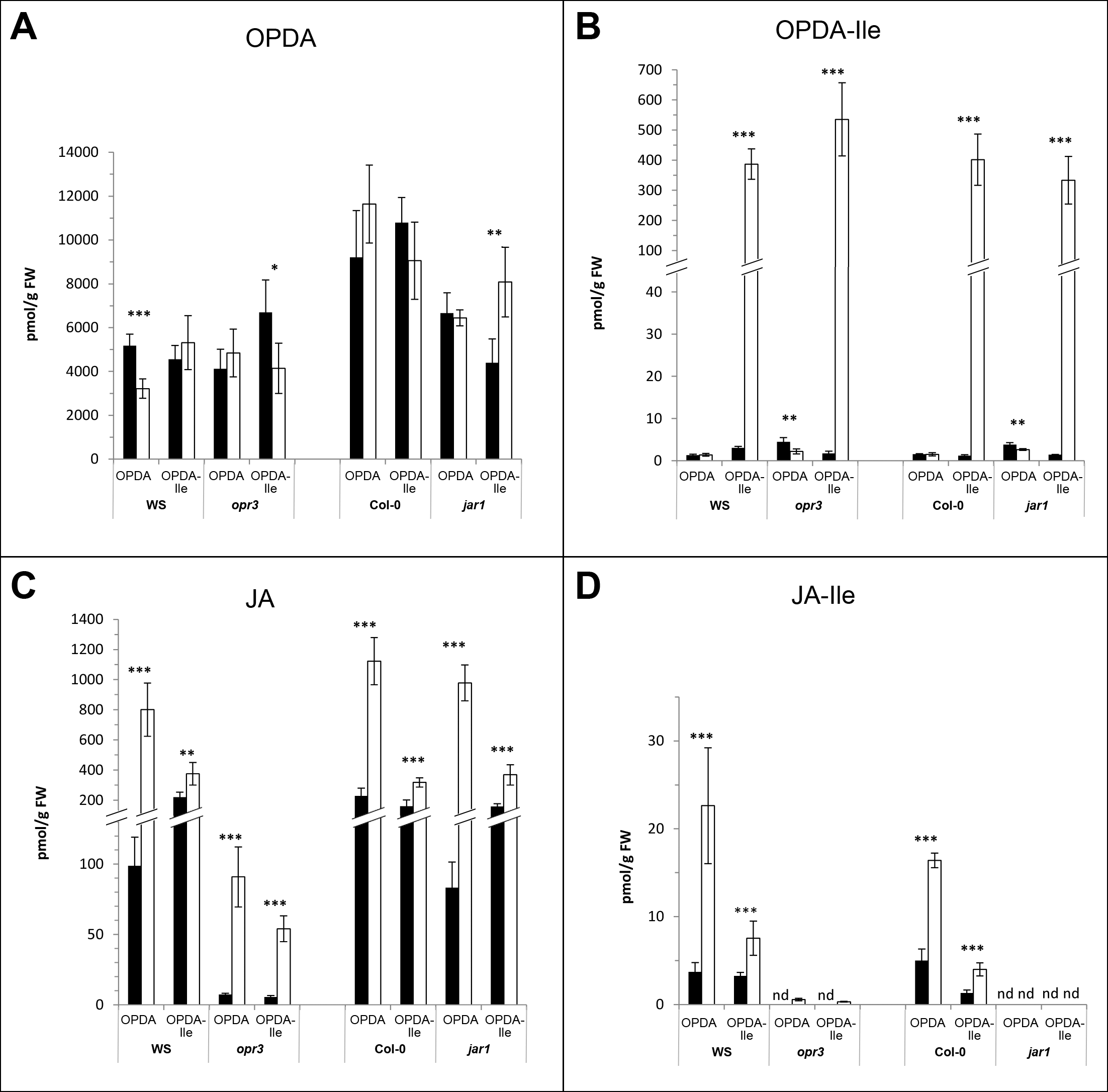

Supplement: S1 Fig — Leaves of plants grown for 6 weeks under long-day conditions were floated on 30 μM OPDA or 30 μM OPDA-Ile for 30 min (white bars). Two independent controls (black bars) were performed by treatment with bi-distilled H2O containing 0.87% [v/v] and 0.56% [v/v] acetonitrile, respectively. Compounds were quantified according to Floková et al. (2016). Each value is represented by the mean of five independent biological replicates ± SD. Treatments and controls were pairwise compared by the Student’s t-test, *p≤0.05, **p≤0.01, ***p≤0.001. (TIF) [file pone.0162829.s001.tif]

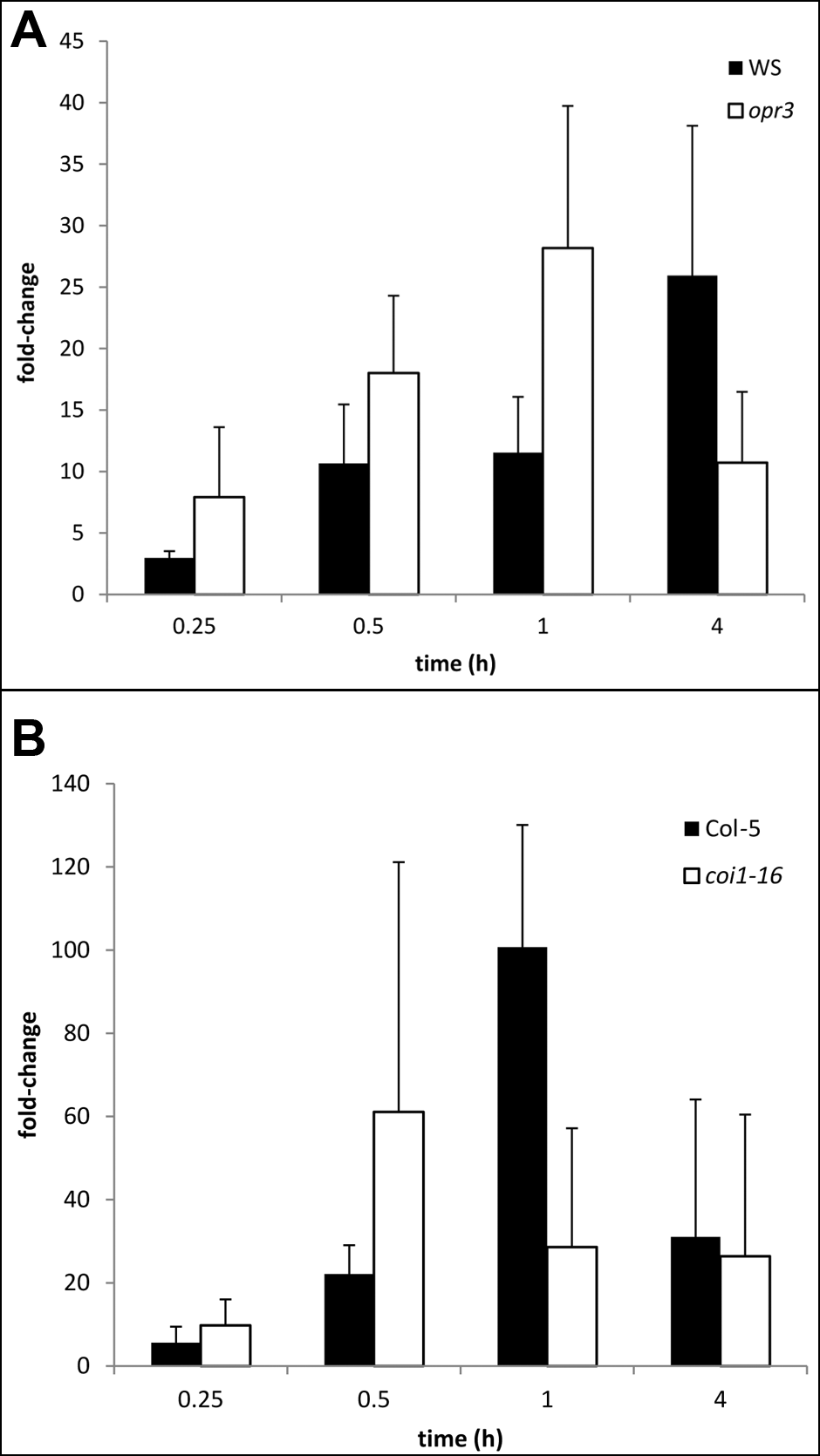

Supplement: S2 Fig — 9-days-old seedlings grown in liquid culture were treated with 50 μM OPDA for the time periods indicated. Controls treated with the solvent acetonitrile (final concentration 0.87% [v/v]) for the same time periods showed constant transcript levels. Fold-change of relative transcript accumulation (ΔΔCt) was determined by qRT-PCR using AtPPA2 as reference and setting t = 0 to 1. Each value is represented by the mean of three independent biological replicates ± SD. Mutants and their respective wild type were pairwise compared by the Student’s t-test and revealed no significant differences. (TIF) [file pone.0162829.s002.tif]

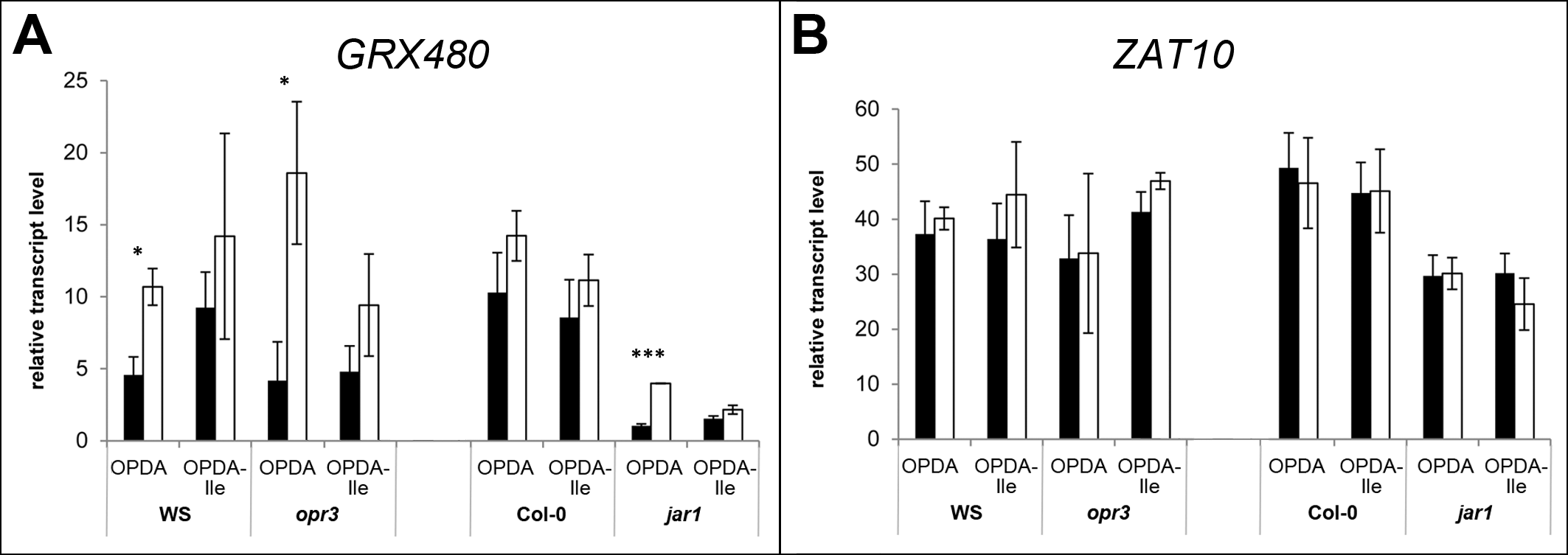

Supplement: S3 Fig — Leaves of plants grown for 6 weeks under long-day conditions were floated on 30 μM OPDA or 30 μM OPDA-Ile for 30 min (white bars). Two independent controls (black bars) were performed by treatment with bi-distilled H2O containing 0.87% [v/v] and 0.56% [v/v] acetonitrile, respectively. Relative transcript levels were quantified by qRT-PCR using AtPP2A as reference. Each value is represented by the mean of three independent biological replicates ± SD. Treatments and controls were pairwise compared by the Student’s t-test, *p≤0.05, **p≤0.01, ***p≤0.001. (TIF) [file pone.0162829.s003.tif]
